# Novel Immortal Cell Lines Support Cellular Heterogeneity in the Human Annulus Fibrosus

**DOI:** 10.1371/journal.pone.0144497

**Published:** 2016-01-21

**Authors:** Guus G. H. van den Akker, Don A. M. Surtel, Andy Cremers, Stephen M. Richardson, Judith A. Hoyland, Lodewijk W. van Rhijn, Jan Willem Voncken, Tim J. M. Welting

**Affiliations:** 1 Department of Orthopaedic Surgery, Maastricht University Medical Centre, Maastricht, the Netherlands; 2 Department of Molecular Genetics, Maastricht University Medical Centre, Maastricht, the Netherlands; 3 Centre for Tissue Injury and Repair, Institute of Inflammation and Repair, The University of Manchester, Manchester, United Kingdom; Wayne State University School of Medicine, UNITED STATES

## Abstract

**Introduction:**

Loss of annulus fibrosus (AF) integrity predisposes to disc herniation and is associated with IVD degeneration. Successful implementation of biomedical intervention therapy requires in-depth knowledge of IVD cell biology. We recently generated unique clonal human nucleus pulposus (NP) cell lines. Recurring functional cellular phenotypes from independent donors provided pivotal evidence for cell heterogeneity in the mature human NP. In this study we aimed to generate and characterize immortal cell lines for the human AF from matched donors.

**Methods:**

Non-degenerate healthy disc material was obtained as surplus surgical material. AF cells were immortalized by simian virus Large T antigen (SV40LTAg) and human telomerase (hTERT) expression. Early passage cells and immortalized cell clones were characterized based on marker gene expression under standardized culturing and in the presence of Transforming Growth factor β (TGFβ).

**Results:**

The AF-specific expression signature included *COL1A1*, *COL5A1*, *COL12A1*, *SFRP2* and was largely maintained in immortal AF cell lines. Remarkably, TGFβ induced rapid 3D sheet formation in a subgroup of AF clones. This phenotype was associated with inherent differences in Procollagen type I processing and maturation, and correlated with differential mRNA expression of Prolyl 4-hydroxylase alpha polypeptide 1 and 3 (*P4HA1*,*3*) and Lysyl oxidase (*LOX*) between clones and differential P4HA3 protein expression between AF cells in histological sections.

**Conclusion:**

We report for the first time the generation of representative human AF cell lines. Gene expression profile analysis and functional comparison of AF clones revealed variation between immortalized cells and suggests phenotypic heterogeneity in the human AF. Future characterization of AF cellular (sub-)populations aims to combine identification of additional specific AF marker genes and their biological relevance. Ultimately this knowledge will contribute to clinical application of cell-based technology in IVD repair.

## Introduction

The intervertebral disc consists of the central nucleus pulposus (NP), which is encircled by the ligamentous annulus fibrosus (AF), and bordered by two flanking (superiorly and inferiorly) cartilaginous end plates (CEPs). The AF is a fibrous tissue with distinct layers of highly oriented Collagen fibers (lamellae) that run in alternating directions at an angle of approximately 30 degrees [1]. The unique structure and composition of the AF contains the compressed NP and, in addition, allows the spine to cope with bending and torsional forces [2]. Further morphological distinction is made between the inner and outer AF layers. The extra cellular matrix (ECM) of the outer AF mainly consists of fibrillar Collagens such as Collagen type I, while the inner AF contains lower amounts of Collagen type I [3]. A gradual increase of Collagen type II and Proteoglycan expression towards the nucleus pulposus further distinguishes the inner AF from the outer AF [3]. The AF is firmly attached to both CEPs [4]. From a clinical perspective, the NP has received considerable attention, as the degenerative processes that accompany disc pathologies are first clinically detected in the NP [5]. However, early pathologies including disc herniation crucially depend on AF integrity. Recent studies indicate that disc herniation does not correlate well with NP degeneration, suggesting that additional factors are involved, including specific aspects of cell biology in both the NP and the AF [5, 6].

Cell density in the AF and NP are low compared to other tissues (± 9000 and 3000 cells/mm^3^, respectively) [7]. Cells in the AF originate from sclerotome-derived mesenchymal cells and are often referred to as chondrofibroblast-like cells, based on morphological characteristics [8]: the outer AF contains cells with an elongated (fibroblast-like) morphology, whereas cells of the inner AF cells display a more rounded (chondrocyte-like) morphology [9]. It is currently unclear whether these cells represent developmentally distinct lineages or whether the local micro-environment determines morphologically and/or functionally divergent phenotypes. It has been suggested that NP cells are recruited to the inner AF and produce collagen type II and Proteoglycans [10], although this idea has been contradicted by recent lineage tracking studies in mice [11, 12]. In analogy with this, morphological conversion of fibroblast-like toward chondrocyte-like cells in the inner AF was described during embryonic AF development in the rat [13] and furthermore the understanding of AF cell heterogeneity is limited [14]. Isolated primary AF cells from healthy or degenerate discs have been used to assess cellular responses to growth factors, inflammatory stimulation and mechano-transduction [15–17]. In addition, such primary cell isolates have been used in combination with biomaterials for disc tissue engineering [18, 19]. Since it is currently unclear how distinct AF cell phenotypes contribute to disc homeostasis and disease, such studies lack functional definition. Establishment of functionally diverse AF cell lines represents a relevant experimental approach to study cell heterogeneity in the AF.

The cells that compose the AF represent a crucial component of the healthy IVD and are involved in disc pathology [20]. Therefore, a detailed understanding of the native AF cell population is imperative to acquire full insight into their involvement in disc pathology and a thorough knowledge of the AF cell population was recently defined as one of the main challenges for successful AF repair [21]. As such we here aimed to generate immortal clonal cell models to enhance studies of the human AF cell population and to test the idea that phenotypically distinct AF cell populations can be isolated and immortalized.

## Materials and Methods

### Isolation of intervertebral disc cells, cell culture and immortalization

Briefly, non-degenerative healthy disc material was obtained as surplus material from correction surgery (MUMC, Medical Ethical Review Committee approval 08-4-021; by Dutch law, informed patient consent is part of the MERC approval and not required separately from next of kin, caretakers, or guardians on behalf of others; all consent was based on the approval obtained from the local MERC; see Table 1). To obtain primary AF cell isolates, AF tissue was collected during scoliosis correction surgery performed on two young individuals (donor 1: D1; donor 2: D2; Table 1). Tissue samples were macroscopically dissected in AF and NP tissue with omission of the inner AF and transition zone. Isolation of IVD cells was described in detail elsewhere [22]. Cells were cultured in maintenance / control medium (Control: DMEM-F12/Glutamax (Gibco), 10% fetal calf serum (FCS; Biowhittaker, cat no DE14-801F), 1% antibiotic/antimycotic (Gibco), 1% non-essential amino acids (NEAA; Gibco)). Initial cell seeding was performed at a density of 30,000 cells/cm^2^ in culture plates (Greiner). Upon confluency (P0) cultures were expanded as “pools” (1:2 to P5) to obtain sufficient material for initial characterization. A detailed description of the immortalization protocol and proof of immortalization was described previously [22]. Briefly, immortalized cell pools were generated from P5 cells by serial transduction of primary cells with retroviral particles carrying coding sequences for SV40LTAg and human telomerase (hTERT). Single cell clones were derived by limiting dilution and cell clones were expanded individually. After immortalization and clonal expansion D2-derived cells showed a proliferation rate more similar to that of the parental lines [22]; for this reason experiments were continued with D2-derived cell clones. When monolayer AF cultures were grown in medium containing TGFβ, 30,000 cells/cm^2^ were plated and incubated the next day (t = 0) with DMEM/F12, 1% antibiotic/antimycotic, 1% Insulin/Transferrin/Sodium Selenite (ITS; Gibco), 50 μg/ml Ascorbic acid-2-Phosphate-deoxycholate (Sigma-Aldrich), 1 ng/ml TGFβ3 (Gibco PHG9305) and 1% NEAA, for the indicated time period. Seeding at this density resulted in a confluent dish one day post-plating. Phase contrast images were taken with a Nikon Eclipse TE200 microscope using available imaging software.

**Table 1:** IVD donor characteristics and clones.

Donor*	gender	age	medical indication	position IVD	AF clones
D1	F	15	idiopathic scoliosis	T7-T10	36
D2	M	13	spina bifida/scoliosis	T6-L1	34

Tissues were obtained from young adolescent scoliosis patients who received correction surgery. In contrast to herniated or adult discs, these intervertebral discs showed no signs of disc degeneration with intact annulus fibrosis tissue surrounding clear lucid nucleus pulposi; both tissues types were macroscopically clearly distinguishable.

*: Donor isolates D1 and D2 in this manuscript correspond to donors D4 and D5, respectively; patient characteristics have recently been published [23]. The total number of generated clones is indicated in column “AF clones”.

F = Female

M = Male, age in years at the time of surgery, discs obtained

T = thoracic

L = lumbar intervertebral discs.

### (Immuno)histochemistry of IVD tissue sections

IVD tissue from above correction surgery was decalcified in formalin/EDTA and dehydrated following standard procedures. Next sections were embedded in paraffin. Tissue sections were cut at 5 μm and positioned on Superfrost Plus slides (Thermo Scientific). Prior to histochemistry, sections on slides were deparafinized and rehydrated using standard protocols. Safranin O (0.1%) was used to stain proteoglycans and counterstaining was performed with Fast Green (0.1%). The stained sections were dehydrated and mounted in Histomount (Thermo, Runcorn, Cheshire, UK) for microscopic analysis. For immunohistochemical analysis human IVD tissue was obtained from a deceased donor (45 year old male), with no history of back pain or other relevant clinical history during *post-mortem* examination (written informed consent was obtained from the donor’s relatives and approval for the study was granted by the local ethics committee: North West Research Ethics Committee). Representative tissue biopsies were processed to paraffin wax and immunohistochemical staining performed on 5 μm sections as previously described [23]. Briefly, sections were deparafinized, rehydrated and heat-mediated antigen retrieval performed using 10 mM Tris/1mM EDTA, pH9 at 95°C for 10 minutes in a steamer. Endogenous peroxidase was blocked using 3% hydrogen peroxide in TBS for 1 hr and non-specific binding sites blocked with 25% normal goat serum in TBS for 45 minutes. Sections were incubated overnight at 4°C with rabbit polyclonal primary antibody for P4HA3 (1:100 in 1% BSA in TBS; Sigma, HPA007897). Biotinylated goat anti-rabbit secondary antibody was used, and staining was disclosed using Vectastain Elite ABC Reagent and a diaminobenzidine chromogen. The negative control used the appropriate IgG (Dako) in place of the primary antibody at equal protein concentration. Stained sections were viewed under light microscopy, and images were acquired using an InfinityX camera with DeltaPix software. Alternatively, sections was scanned using the Pannoramic 250 Flash II digital slide scanner (3DHistech®) and visualised using the Pannoramic Viewer software (3DHistech®).

### RNA isolation and quantitative real time PCR

To isolate RNA, cells were disrupted in Trizol (Invitrogen). RNA isolation, RNA quantification (UV)-spectrometry (Nanodrop, Thermo Scientific), and cDNA synthesis were performed as described before [24]. Real-time quantitative PCR (RT-qPCR) was performed using Mesagreen qPCR master-mix plus for SYBR^®^ Green (Eurogentec). Validated primer sets used are depicted in Table 2. An Applied Biosystems ABI PRISM 7700 Sequence Detection System was used for amplification: initial denaturation 95°C for 10 min, followed by 40 cycles of DNA amplification. Data were analyzed using the standard curve method and normalized to *Cyclophillin B (PPIB) mRNA levels*.

**Table 2:** Quantitative PCR primer list.

symbol	gene name	forward primer	reverse primer
*ACAN*	Aggrecan	GCAGCTGGGCGTTGTCA	TGAGTACAGGAGGCTTGAGGACT
*ADAMTS17*	ADAM with thrombospondin type I motif 17	AATTTGGCCTTTACCATCGCC	CGTGGTTCATGCCCAAGTTGT
*BMP1*	Bone morphogenetic protein 1	CCTCCCCTGAA-ACCCCAAT	CCCGGGTGTGACAGAGATG
*COL1A1*	Collagen type I, Collagen α1	TGGAGAGTACTGGATTGACCCC	TGCAGAAGACTTTGATGGCATC
*COL2A1*	Collagen type II, Collagen α1	TGGGTGTTCTATTTATTTATTGTCTTCCT	GCGTTGGACTCACACCAGTTAGT
*COL5A1*	Collagen type V, Collagen α1	GATGTCGCTTACAGAGTCACCAA	AAATGCAGACGCAGGGTACAG
*COL12A1*	Collagen type XII, Collagen α1	TGACAACCCTTTCCGACACA	CTCCTCACGGTTCTAAAATTTGC
*COMP*	Cartilage oligomeric matrix protein	CAAGGCCAACAAGCAGGTTTG	CAGTTATGTTGCCCGGTCTCA
*PPIB*	Cyclophillin B	CCTGCTTCCACCGGATCAT	CGTTGTGGCGCGTAAAGTC
*LOX*	Lysyl oxidase	TAGCCACTATGACCTGCTTGATG	AACTTGCTTTGTGGCCTTCAG
*P4HA1*	Prolyl 4-hydroxylase, alpha polypeptide 1	CCCATTTTGACTTTGCACGG	CCCCAGCTCTTTGAAAGCATC
*P4HA2*	Prolyl 4-hydroxylase, alpha polypeptide 2	TCAAACTGACACCCCGTAGACA	TGTTGCCATGGTGGTACCTACA
*P4HA3*	Prolyl 4-hydroxylase, alpha polypeptide 3	GGAGCCACAGCCTTCATCTATG	ATTCCTAACCACAGGCAGCT
*PLOD1*	Procollagen-lysine, 2-oxoglutarate 5-dioxygenase 1	ATGGCCTTCTGTGCCAACAT	CGGTTGGTCAGGAACATGAAC
*PLOD2*	Procollagen-lysine, 2-oxoglutarate 5-dioxygenase 2	AGCGTTCTCTTCGTCCTCATCA	TTTGCAACCACCTCCCTGAA
*PLOD3*	Procollagen-lysine, 2-oxoglutarate 5-dioxygenase 3	TTTCCCGGTTACCACACCAAG	CCGGTAGCGAACCACAAAGTT
*SFRP2*	Secreted frizzled-related protein 2	TGTGCCACGGCATCGA	TCGTGGCCCAGCAGGTT
*SOX9*	SRY-box 9	AGTACCCGCACCTGCACAAC	CGCTTCTCGCTCTCGTTCAG
*TLL1*	Tolloid-like 1	ATTCTCCCCTCCCGTGATG	GACGGGTTCGCTGACCAAT

Validated real time primer sets for the indicated genes that were subjected to quantitative PCR analysis in this study.

### Protein extraction and immunoblotting

Protein extraction and immunoblotting were performed and analyzed as described previously with minor adjustments [25]. For extraction, cells were lysed in RIPA buffer (50 mM Tris-HCl pH 8.0, 150 mM NaCl, 0.1% SDS, 5 mM EDTA, 0.5% w/v Sodium Deoxycholate, and 1% NP-40) supplemented with protease and phosphatase inhibitors (Roche). Lysates were sonicated on ice using the Soniprep 150 MSE at amplitude 10 for 14 cycles (1 second on/1 second off). Insoluble material was removed by centrifugation (10 minutes, 16000x g, 4°C). Protein concentration was determined using a BCA protein assay kit (Pierce/Thermo Fisher Scientific). Proteins samples were separated by SDS-PAGE and transferred to Nitrocellulose membranes (Protran BA-8). Membranes were blocked (1 hour, 5% non-fat dry milk powder (Campina), ambient temp), and incubated with primary antibodies (overnight, 4°C). Antisera: polyclonal goat anti-COL1A1 (1310–01; Southern Biotech), mouse monoclonal β-Actin (clone C4, 08691001; MP Biomedicals, Santa Ana, CA, USA), mouse monoclonal α-Tubulin (clone B-5-1-2, T6074; Sigma-Aldrich). Secondary antisera: polyclonal rabbit anti-goat (P0449; Dako Cytomation, Glostrup, Denmark), rabbit anti-mouse (P0260; Dako). Signals were detected using enhanced chemoluminescence. X-ray films were scanned and analyzed with Quantity One (Biorad).

### Cell assays (proliferation, DNA)

At each indicated time point cells were washed and fixed (3.7% formaldehyde/PBS, 10 minutes, ambient temperature), rinsed with demineralized water, stained (0.1% Crystal violet, 30 minutes, ambient temperature) and washed with demineralized water. Crystal violet was extracted with 10% acetic acid; absorbance was determined at 590 nm (Benchmark, Biorad).

### Statistics

**Fig 1:**
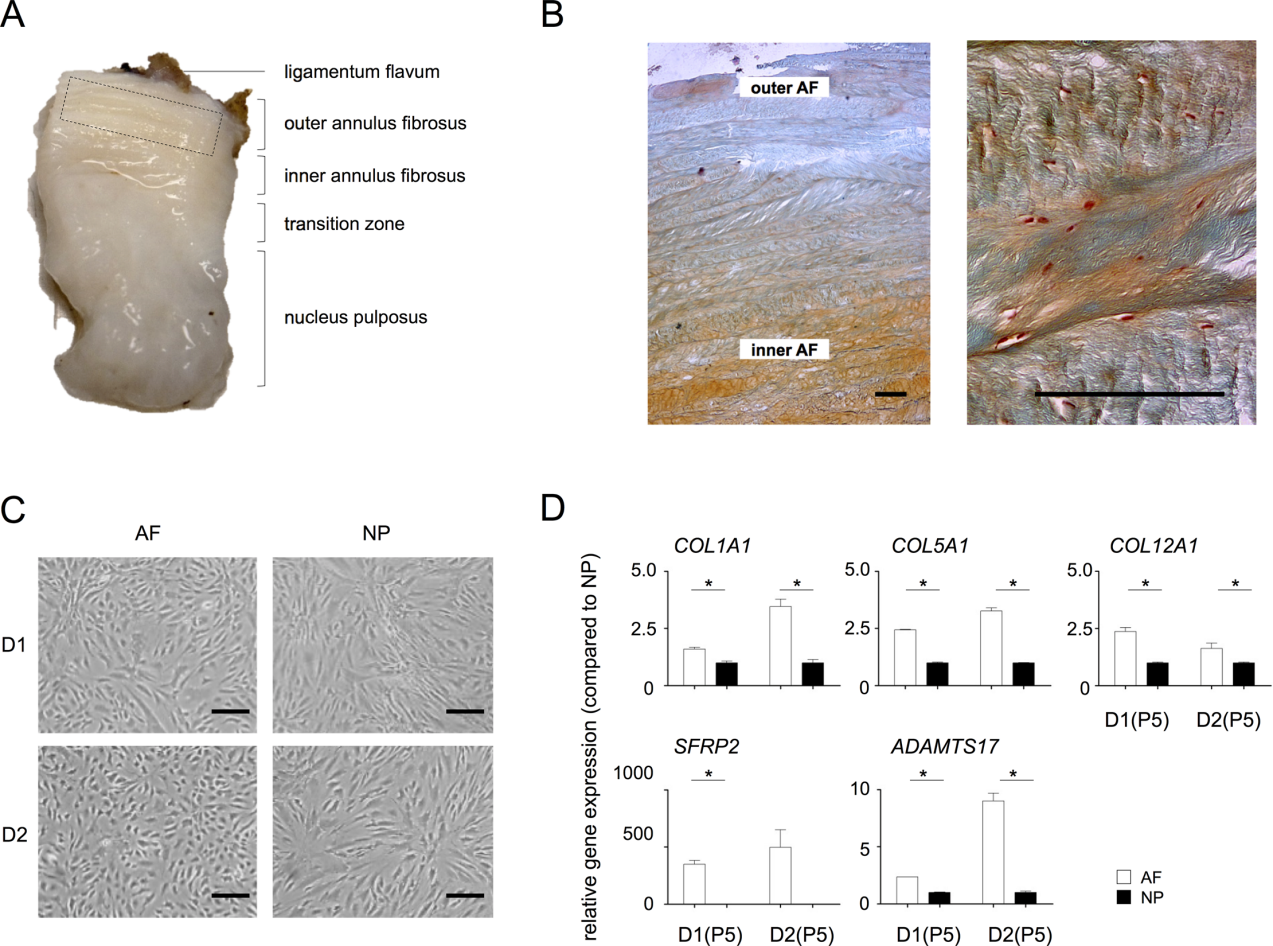
Confirmation of AF cell phenotype *in vitro*. **A)** Representative image of a human IVD tissue specimen from a scoliosis donor. The relevant tissue zones are indicated; the dotted box indicates, by approximation, the tissues section used for AF cell isolation. **B)** Safranin O/Fast Green staining of AF tissue from a scoliosis donor at 25x (left image) and 100x magnification (right image). Bars indicate 200 μm. **C)** Phase-contrast images of primary NP and AF cell pools from donor 1 (D1) and from donor 2 (D2) at passage 5 (P5). Primary AF cells were slightly more rounded, while NP cells showed a typical wave-like patterning. Bars indicate 20 μm. **D)** Gene expression analysis of the putative AF markers *COL1A1*, *COL5A1*, *SFRP2*, *COL12A1* and *ADAMTS17* in primary AF (white bars) and NP (black bars) cell isolates from donor 1 D1(P5) and donor 2 D2(P5), respectively; gene expression was normalized to *Cyclophillin B* mRNA levels and data is presented relative to expression in NP cells. Statistical significance was assessed by Student’s *t*-test * p<0.05.

**Fig 2:**
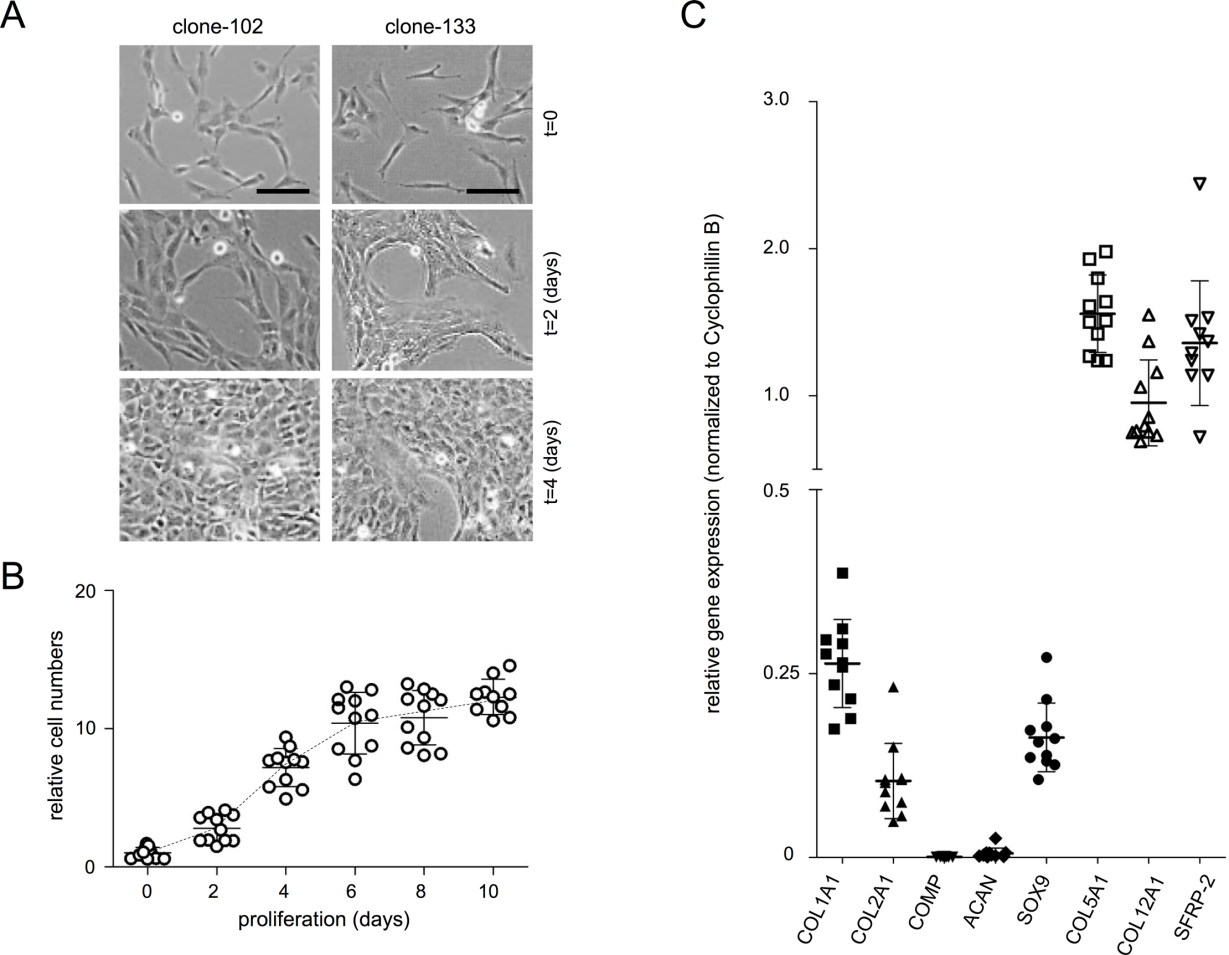
Clonal phenotypes of AF cell lines. **A)** Phase-contrast images of two representative AF cell clones 102 and 133 (D2) under normal, (*i*.*e*. non-differentiation) maintenance conditions. Cell clones showed preferential cell-cell contacts and alignment at 1–2 days post-plating. Time points in days are indicated after seeding 6,400 cells/cm^2^ at day zero; the t = 0 image represents 12 hours post-plating. Bars indicate 20 μm. **B)** Spectrometric quantification of Crystal violet staining assays, reflecting growth of eleven immortalized cell clones. Every dot represents the average value of a biological triplicate measurement for a single clone. Mean values and standard deviations are depicted for eleven independent clones. At approximately 6 days post-plating (P10), a plateau was reached due to confluence of the cell cultures. **C)** Gene expression analysis of *COL1A1*, *COL2A1*, *COMP*, *ACAN*, *SOX9* and the novel AF markers *COL5A1*, *COL12A1*, *SFRP2*. Every dot represents a single clone and shows the average of a biological triplicate measurement. Mean expression values and standard deviations are depicted per marker gene for eleven clones. Gene expression values were normalized to *Cyclophillin B* mRNA levels.

**Fig 3:**
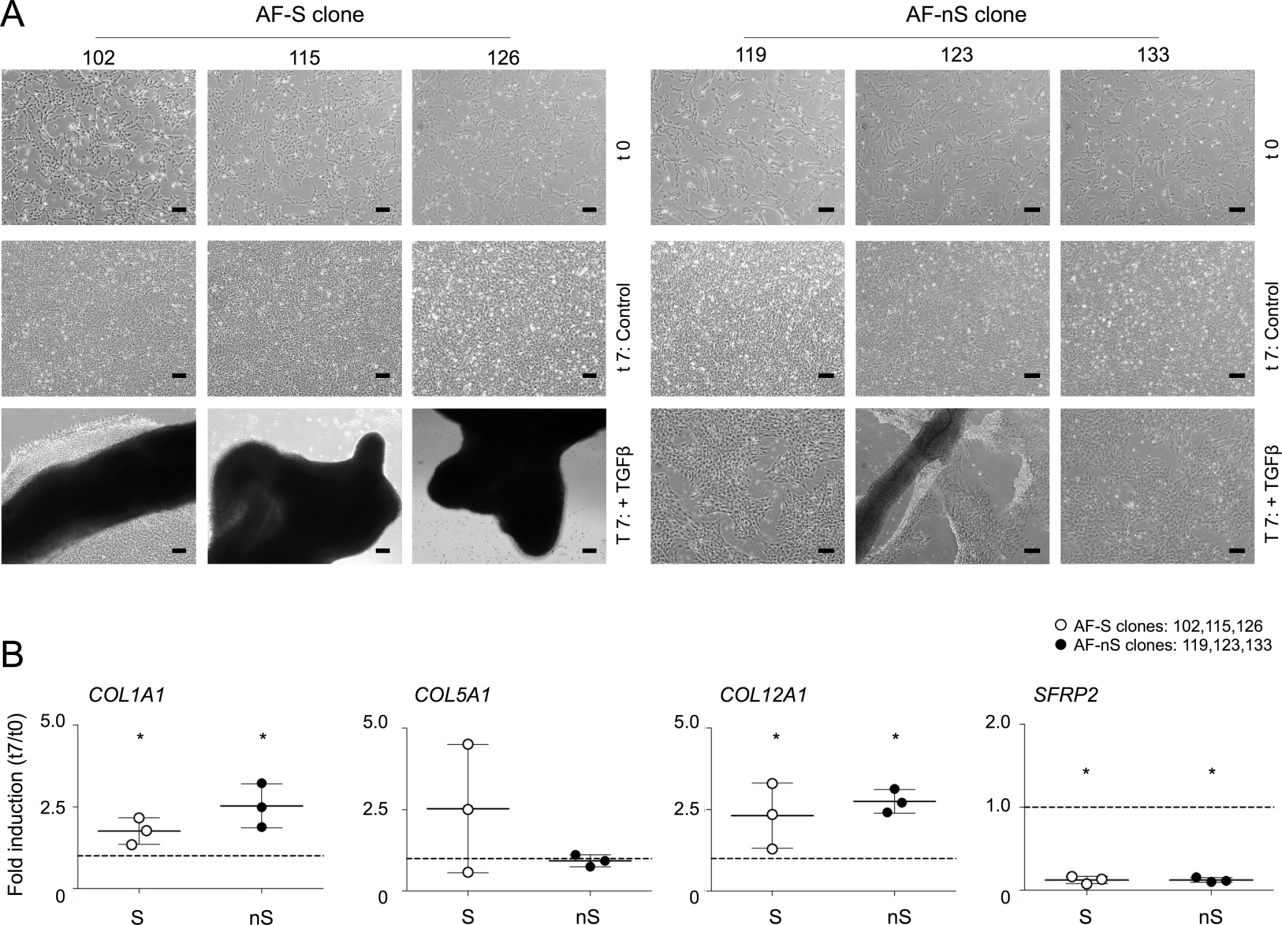
TGFβ3-induced sheet formation in a subgroup of AF clones. **A)** Phase contrast images of AF-S clones 102, 115, 126 and AF-nS clones 119, 123, 133 (from D2) at t = 0 and cultured in control medium (Control) or TGFβ3 containing medium (+ TGFβ3) for 7 days. Bars represent 20 μm. Cells did not exhibit sheet formation in control medium. **B)** Gene expression analyses of *COL1A1*, *COL5A1*, *COL12A1* and *SFRP2* in immortal AF cell clones. Every dot represents a single clone and is the average of a biological triplicate measurement. Gene expression was normalized to *cyclophillin B* mRNA levels. Fold induction (t = 7 TGFβ3 / t = 0) was calculated for each clone separately. Mean and standard deviations are depicted for the three clones together per gene. Statistical significance was assessed by Student’s *t*-test; * p<0.05.

**Fig 4:**
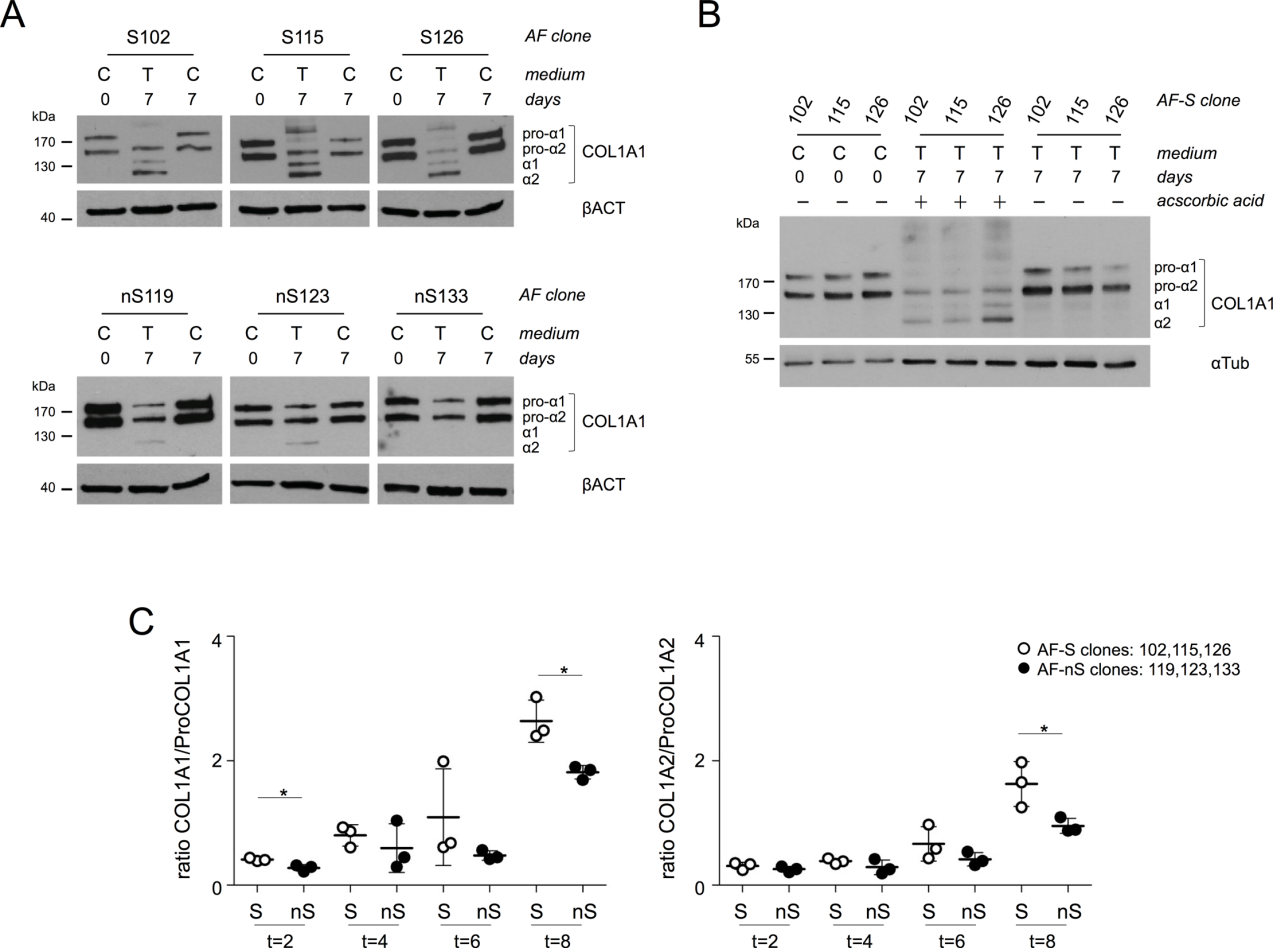
Collagen processing determines the sheet forming capacity in AF clones. **A)** Immunoblot analyses of Collagen type I protein in AF-S and AF-nS clones cultured in control or TGFβ3 containing medium for 7 days. Procollagen-alpha 1 (180 kDa; pro-α1) and Procollagen alpha 2 (145 kDa; pro-α2) variants of Collagen type I are indicated. The appearance of mature alpha 1 (135 kDa; α1) and alpha 2 (120 kDa; α2) variants of Collagen type I correlated well with sheet formation in AF-S clones. β-Actin (βACT) was used as loading control. **B)** Immunoblot analyses of Collagen type I protein in AF-S clones cultured in TGFβ3 medium with or without ascorbic acid for 7 days. Alpha-tubulin (αTUB) was used as loading control. **C)** Quantification of Collagen maturation as a function of time in AF-S and AF-nS clones. The ratio of mature COL1A1 over ProCOL1A1 and mature COL1A2 over ProCOL1A2 are depicted in the left and right graphs, respectively. At t = 0 no mature forms of Collagen type I were detectable. Statistical significance was assessed by Student’s *t*-test; * p<0.05.

**Fig 5:**
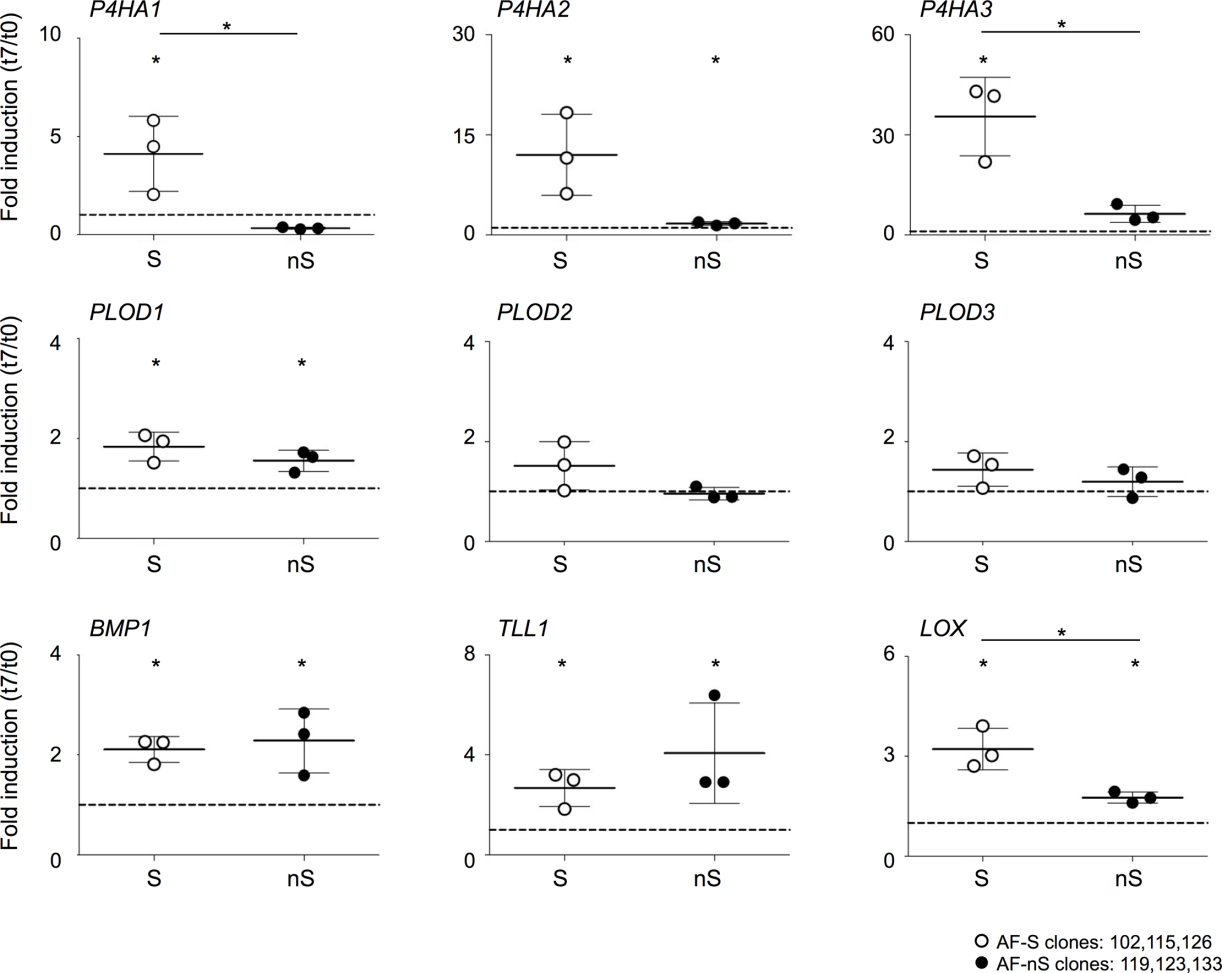
Collagen modifying enzymes are transcriptionally regulated by TGFβ3 in sheet forming AF clones. Upper panels: expression analysis of *P4HA1-3* genes in AF-S and AF-nS clones at t = 0 and t = 7 days of culturing in TGFβ3. Middle panels: expression analysis of *PLOD1-3* genes in AF-S and AF-nS clones at t = 0 and t = 7 days of culturing in TGFβ3. Lower panels: expression analysis of genes involved in cleavage of Collagen type I pro-peptides (*BMP1*, *TLL1*) and crosslinking of Collagen fibers (*LOX)* in AF-S and AF-nS clones at t = 0 and t = 7 days of culturing in TGFβ3. Gene expression was normalized to *cyclophillin B* mRNA levels. Fold induction (t = 7 TGFβ3 / t = 0) was calculated for each clone separately. Mean and standard deviations are depicted for the three clones together per gene. Statistical significance was assessed by Student’s *t*-test; * p<0.05.

Statistical significance (p < 0.05) was determined by two-tailed student *t* tests. To test for normal distribution of input data, D’Agostino–Pearson omnibus normality tests were performed. All quantitative data sets presented passed the normality tests. In Figs 1 and 2 a two-tailed student *t* test was used and in Figs 3, 4 and 5 a one-tailed student *t* test was used as only a positive difference was expected. Gene expression analyses show mean and standard deviation.

## Results

### AF cell isolation and basic AF cell characteristics

Tissue from two independent young donors that was clearly distinguishable as outer AF (Fig 1A; Table 1) was carefully selected and dissected for this purpose, to avoid cell contamination by other IVD tissue types (*i*.*e*. inner AF/transition zone, NP). Safranin-O/Fast Green staining of paraffin sections from tissue samples confirmed that the selected AF tissue displayed typical AF morphology: radial layers of alternately oriented Collagen fibers, cells of elongated morphology aligned parallel to the fibers and increasing glycosaminoglycan content toward the NP (Fig 1B).

Cells from dissected AF tissues were enzymatically released from their ECM and allowed to adhere to culture plates. AF cell morphology in monolayer cultures was similar to previous reports [26] and primary AF and NP cell cultures showed consistent morphological features (primary AF cells were slightly more rounded, while NP cells showed a typical wave-like patterning), independent of donor (Fig 1C). We aimed to confirm tissue of origin prior to immortalization by measuring expression of putative AF markers; candidate markers were selected based on highest fold differential expression and on marker overlap among these studies [27–30]. NP cells that had been isolated simultaneously from adjacent NP tissue were used in comparative expression studies to independently establish distinctive *in vitro* morphology and gene expression phenotypes. Expression of most previously reported AF marker genes *COL1A1* [27, 30], *COL5A1* [27] and *COL12A1* [30] was at least 2 fold higher in primary AF cultures of two independent donors as compared to matched NP cultures at passage 5 (P5) (Fig 1D). The putative AF marker *SFRP2* [29] was exclusively expressed in primary AF cells (Fig 1D). In addition, we found differential expression of *ADAMTS17*: NP cells consistently expressed lower *ADAMTS17* levels (Fig 1D). NP-specific marker expression analysis was published elsewhere [22]. This initial data thus confirms distinctive tissue of origin of primary AF and NP cells.

### Cell line generation and characterization of AF cell clones

A total of 70 cell clones (Table 1) were obtained from immortalized P5 cells that displayed a comparable fibroblastic morphology (Fig 2A), in agreement with published reports [31, 32]. Eleven randomly chosen clones showed nearly similar proliferation rates with an average population doubling time (PDL) of 50.71 hours in the exponential phase (Fig 2B).

To evaluate whether cell clones retained an AF-specific marker expression profile, we measured expression of genes associated with a chondrocyte-like phenotype: *COL2A1*, *SOX9* and *ACAN* [33]. In addition we evaluated the articular chondrocyte marker COMP, which was not expressed in the NP or the AF [27]. A ratio 800:1 of *COL2A1* over *COL1A1* mRNA has previously been established as a measure to differentiate between NP and AF cells [34]. Immortal AF cell clones indeed showed higher levels of *COL1A1* mRNA compared to *COL2A1* mRNA (Fig 2C; average ratio 2.5:1). *SOX9* mRNA levels were relatively low, yet detectable in all tested clones, which is in accordance with an earlier publication [35]. *COMP* and *ACAN mRNA* levels were very low/absent, in good agreement with an AF cell phenotype [36]. We evaluated whether primary AF marker gene expression profiles, established in Fig 1C, were retained in immortal AF cell clones. The AF markers *COL1A1*, *COL5A1*, *COL12A1* and *SFRP-2* were expressed in AF cell clones, except for *ADAMTS17* (Fig 2C). Absolute mRNA expression levels of our defined set of AF markers (*COL5A1*, *COL12A1*, *SFRP2*) were higher than *COL1A1*, *COL2A1*, *ACAN* and *SOX9* mRNA levels (Fig 2C). Although morphological differences among clones were not readily discernable, marker gene expression levels were relatively heterogeneous between cell clones (Fig 2C).

### Functional differences between AF cell clones

Transforming growth factor beta 3 (TGFβ3) is a well-known morphogen used to induce ECM synthesis of AF cells [37]. We previously showed that a medium containing TGFβ3 induced divergent responses in primary AF and NP cells [22]. Therefore the AF cell clones were cultured in this TGFβ containing medium. We observed the formation of three-dimensional ECM structures within seven days after exposure to TGFβ (Fig 3A). Importantly, a subset of clones: AF-102, AF-115 and AF-126 formed a 3D cellular “sheet” within seven days, whereas clones AF-119, AF-123 and AF-133 showed dissimilar sheet formation and contraction dynamics within this timeframe. Based on the differential dynamics in sheet formation, AF clones will be referred to as AF-S (sheet-forming) and AF-nS (non sheet-forming) from hereon. As sheet formation *in vitro* has been associated with Collagen formation [38], mRNA levels of Collagens *COL1A1*, *COL5A1* and *COL12A1* mRNA were determined (Fig 3B). Basal expression levels of *COL1A1*, *COL5A1* and *COL12A1* mRNA were not different between the two groups of subclones, *COL5A1* exempted: 2 out of 3 representative AF-nS clones expressed higher basal *COL5A1* levels. Expression of the *COL1A1* and *COL12A1* markers was increased as a result of seven days exposure to TGFβ3 in both AF-S and AF-nS clones. *COL5A1* mRNA expression was higher in 2 out of 3 clones in AF-S clones, whereas overall expression levels in AF-nS had not changed. Of note, *SFRP2*, a previously reported putative AF marker, was strongly down regulated in all clones under these conditions. Thus, although differences in sheet formation were evident between clones, expression of Collagen genes *per se* did not explain the divergent ability to form 3D sheets.

The macroscopically detectable formation of contracting Collagenous cell sheets indicated that Collagen maturation may be involved in sheet formation, as has been reported in other studies [38]. The formation of clearly visible fibers in phase-contrast images precedes contraction of cell sheets. We therefore tested for potential differences in Collagen maturation between the AF clones. COL1A1 maturation and processing was visualized by immunoblotting. COL1A1 protein appears in two forms: alpha1 and alpha2. Two alpha 1 chains and one alpha 2 chain form one Collagen type I triple-helical molecule [39]. The N- and C-terminal domains in Procollagen prevent fiber formation upon assembly of Collagen molecules. Collagen molecules undergo various post-translational modifications during maturation and processing [39]. Upon secretion into the ECM, these pro-domains are enzymatically removed to allow fiber formation [39]. The ascorbic acid-dependent formation of hydroxylated proline residues stabilizes the Collagen molecule and propels formation of larger Collagen networks [40, 41]. Immunoblotting revealed clear maturation of Collagen type I alpa1 and -alpha2 in clones AF-102, AF-115 and AF-126 cell lysates grown in the presence of TGFβ3 (Fig 4A). Within the same time-frame, fragments corresponding to mature Collagen molecules could not be detected in clones AF-119, AF-123 and AF-133 (Fig 4A). Thus, differential sheet formation in the presence of TGFβ3 correlated with COL1A1 maturation. To determine whether the observed effects depended on ascorbic acid-mediated formation of mature Collagen type I, AF-S clones were grown in the presence of TGFβ3 in medium containing ascorbic acid or not. Relevantly, TGFβ3-induced Procollagen maturation does not occur in AF-S clones in the absence of ascorbic acid (Fig 4B). Consistent with this observation, sheet formation did not occur in the absence of ascorbic acid (data not shown).

Differential processing of Procollagen type I (ProCOL1) in the different subsets of AF clones was quantified in a more detailed time course experiment. Three representative clones for each group were exposed to TGFβ3 for 0, 2, 4, 6 or 8 days. To obtain additional evidence for a divergent ability to process Collagens, ratios of mature/Procollagen were determined for COL1A1 and COL1A2 as a function of time by immunoblotting and subsequent densitometry. Procollagens began to be processed within 2 days of culturing in the presence of TGFβ3, as they became detectable by immunoblotting at this time point (data not shown). Within 4 and 6 days of culturing, a clear trend towards significantly different COL1A1/ProCOL1A1 ratios between AF-S and AF-nS became apparent (Fig 4C). At 8 days of TGFβ3 exposure, the COL1A1/ProCOL1A1 (Fig 4C, left panel) and COL1A2/ProCOL1A2 (Fig 4C, right panel) ratios were substantially increased (as compared to t = 2) and significantly higher in AF-S clones, compared to AF-nS clones (Fig 4C). This collective data thus far suggests that Collagen type 1 maturation and processing in AF-S clones is strongly associated with a more rapid sheet formation and detachment than in AF-nS clones.

To further examine the origin of the differential Procollagen processing observed between AF-S and AF-nS clones, we measured the expression of genes encoding proteins involved in Procollagen processing- and post-translational modification of Collagen. The enzymes Prolyl 4-hydroxylase alpha polypeptide 1, 2 and 3 (P4HA1-3) are responsible for the formation of hydroxyproline groups on Collagen molecules in the endoplasmatic reticulum (ER) [42]. Consistent with enhanced Collagen processing, AF-S cell clones showed an increased expression of *P4HA1*, *P4HA2 and P4HA3* of 4, 6 and 10 fold, respectively (Fig 5, top panels). In sharp contrast, *P4HA1-3* gene expression was non-responsive to TGFβ3 in AF-nS clones. Prolyl hydroxylase gene expression thus correlated strongly with sheet forming capacity in AF-S cells. The Procollagen-lysine, 2-oxoglutarate 5-dioxygenase 1, 2 and 3 (*PLOD1-3*) enzymes catalyze the hydroxylation of lysyl residues of Collagen peptides in the ER and these residues are critical for the formation of intramolecular crosslinks. The expression levels of *PLOD1* was significantly increased in both AF-S and AF-nS clones under TGFβ3 conditions; all three *PLOD* genes showed a trend toward higher expression in AF-S clones (Fig 5, middle left panel). Subsequently we measured two genes involved in extracellular Procollagen cleavage. Tolloid-like 1 (TLL1) and Bone Morphogenic Protein-1 (BMP-1) are responsible for extra-cellular C-terminal Procollagen cleavage, while ADAMTS2 is involved in N-terminal cleavage. Both *TLL1* and *BMP1* expression was significantly induced in AF-S and AF-nS clones by TGFβ3 containing medium at day 7 (Fig 5, bottom panels). *ADAMTS2* expression did not show any (differential) response to TGFβ3 (data not shown). Absence of consistent expression differences between AF-S and AF-nS for these genes suggests that these genes may not play a significant role in the observed divergent sheet formation capacity between AF subclones. Lysyl oxidase (LOX) is located in the extracellular environment and involved in covalent crosslinking of Collagen networks. *LOX* expression was significantly induced in AF-S (average 5 fold) and decreased in AF-nS cell clones (2 fold). *LOX* expression was significantly different between AF-S and AF-nS clones at t = 7. The clearly significant fold change between AF-S and AF-nS clones for the genes *P4HA1*, *P4HA3*, *PLOD1* and *LOX* indicate an inherent difference in the ability of AF cell clones to process Procollagen molecules.

**Fig 6:**
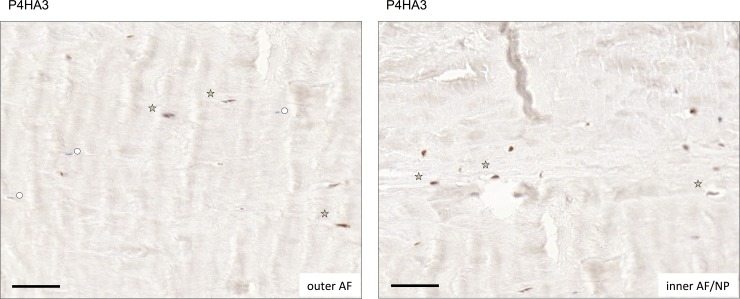
P4HA3 positivity in AF tissue is heterogeneous. Expression of P4HA3 was determined immunohistochemically in human AF tissue. Representative images of outer AF (left panel) and inner AF (right panel) from a 45 year old male donor with no signs of disc degeneration. Asterisks: representative examples of immune-positive cellsl circles: immune-negative cells. Black bars represent 50 μm.

Finally we sought evidence for P4HA3 marker expression at the protein level *in vivo*. Immunohistochemical staining of IVD tissue showed fibroblastic AF cells staining positive for P4HA3 in both the inner and outer AF (Fig 6). Importantly not all cells were positive, particularly in the outer AF, where cells with similar morphology did not detectably express P4HA3. Thus the data presented herein supports inherent cell heterogeneity or different cell states in the native human AF.

## Discussion

In this study we aimed to generate the first stable *in vitro* cell models representing the human AF. First, we established an AF gene expression signature that discriminates cultured primary AF cells from primary NP cells. Subsequently, we immortalized AF cell pools from two independent donors and generated a total of seventy clonal cell lines. We characterized eleven random AF cell clones based on the AF gene signatures and a number of additional genes routinely employed in IVD research. The immortal cell clones maintained an AF phenotype which was largely comparable to primary AF cells. Using medium containing TGFβ3 [37] [43] that was previously used to differentiate primary AC and NP cells [22, 24], we were able to differentiate between two subsets of immortal AF clones based on their ability to process COL1A1: AF-S subclones rapidly produced mature Collagen forms that propelled 3D cellular sheet formation and contraction, while a second group of AF clones (AF-nS) did this to a much lower extent. Relevantly, several genes known to be involved in the post-translational modification and processing of Collagen were more prominently induced in AF-S clones. Based on these results, we speculate that our immortalization procedures provides proof for the existence of at least two functionally different cell states in the outer AF that differ in their ability process to Collagen type I. Further research on identifying unique cellular markers may elucidate if these characteristics represent different AF cell types or different (dormant and activated) cell states. Combined with our earlier report on the establishment of functionally distinct NP cell types, our current data support the notion that *in vivo* the AF and NP harbor a heterogeneous cell population.

### AF cell heterogeneity *in vitro* and *in vivo*

The finding that phenotypically distinct AF cell populations exist *in vitro* paves the way for *in vivo* discovery of additional AF cell subtypes. Senescent and non-senescent AF cells are known to be present in the IVD *in vivo* [44, 45]. It is important to note that the retrovius-based immortalization procedure employed is biased toward immortalization of proliferating cells. Hence it is anticipated that not all possible AF cell phenotypes were immortalized, most notably terminally differentiated, non-proliferative cells. SV40LTAg, which interferes with pRB and TP53 function, and hTERT prevent senescent responses. This response may be important for mechanisms leading to disc degeneration. Although SV40LTAg may affect cell phenotype and differentiation characteristics [46], we did not detect differences in proliferation or marker gene expression between the AF cell subtypes reported herein prior to stimulation with TGFβ. Relevantly, cellular phenotypes of neuronal cells are retained by immortalization with SV40LTAg and hTERT [47]. Although we cannot formally rule out an effect of SV40LTAg and/or hTERT on AF cell responses under the culture conditions used herein, the data thus far suggest that the phenotypic variation arises despite immortalization. Future experiments with conditionally immortalized models may further improve the representation of IVD cell lines.

The observed differential capacity of single AF cell clones to process Collagens *in vitro* is an indication that AF cell heterogeneity exists. This is supported by immunohistochemical studies showing diversity in the AF cell morphology and orientation [9, 14]. Two distinct cellular subpopulations, an elongated and a rounded cell type have been described in the embryonic rat AF [9]; it is however not clear how cell morphology relates to Collagen fibrillogenesis at this time point in development. In the bovine AF three cellular subtypes were described: 1) extended cordlike cells that form an interconnected network at the periphery of the disc, 2) cells with extensive, sinuous processes in the inner region of the annulus fibrosus and 3) cells with broad, branching processes specific to the interlamellar septae of the outer annulus [14]. It is conceivable that such cell types differ in their ability to synthesize and or process Collagens. Detailed molecular characterization and marker identification is crucial to elucidate if different cell types exist or that dormant and activated cell states are present.

### AF marker expression

Putative marker genes for AF cells have been reported in several array expression studies of varying tissue origin [27–30]. However most of these reported AF marker genes were not validated by qPCR. *COL1A1* and in particular the ratio of expression with *COL2A1* has been used to discriminate AF from NP cells. We confirmed that *COL1A1*, *COL5A1*, *COL12A1*, *SFRP-2* and *ADAMTS17* expression levels positively discriminate AF from NP cultures. *ADAMTS17* and *COL5A1* were previously found to be expressed in human AF cells [48]. The vast expression difference for *SFRP2* in AF and NP cultures was surprising as this marker appears to be rather unique for AF cultures. Although we find *ADAMTS17* expression in primary AF cells, the expression of this marker was reduced in immortal clones. Although the exact reason for the diminished *ADAMTS17* expression is not known at this moment, it is conceivable that expression of this marker requires the presence of and interaction of cells with a specific AF environment. Alternatively, cells expressing these markers may not have been immortalized. *ADAMTS17*, which encodes a protein involved in Collagen processing, was differentially expressed between AF and NP in primary cells from both donors (3 fold higher in AF). *SOX9* expression was found in all evaluated AF clones. *SOX9* expression is a well described marker for chondrocytes and the expression in every clone, which was derived from a single cell, supports the idea that AF cells harbor a phenotype akin to chondrocyte-like cells. Based on the observation that subcloned (*i*.*e*. single cell–derived) cell lines express these markers, we would propose that the AF does not contain mixtures of chondrocytes and fibroblasts *per se*, but rather that individual AF cells intrinsically harbor expression characteristics of both.

Since there is no clear-cut definition of the AF phenotype, consensus on the morphogens and the conditions required for AF(-like) differentiation is lacking. We utilized a routinely employed culture medium. One important constituent, TGFβ3, is widely used in chondrocyte studies and fibroblasts are often stimulated with this growth factor to study fibrosis [37, 43, 49]. Moreover, we observed that this medium evoked a differential response between AF and NP cultures [22, 24]. Similar to our previously reported observations in NP cell clones, the expression of novel marker genes (e.g. *SFRP2*) was decreased in the presence of TGFβ3 containing medium [22, 24]. The function of these novel marker genes in the IVD has not been established and therefore their down-regulation in response to standard chondrogenic media cannot currently be explained. It is conceivable that TGFβ3 forces these cells to adopt a phenotype *in vitro* which does not occur *in vivo*. Additional comparative studies using different morphogens is required to elucidate optimal AF cell differentiation conditions.

### Collagen maturation as a distinguishing feature among AF subclones

Collagen fiber formation was reported to be dose-dependently regulated by ascorbic acid [50]. Secretion of Collagen is thought to be a rate limiting step in Collagen fibrillogenesis [51]. The inherent difference in the capacity of groups of AF clones to process Collagens is an indication of AF cell heterogeneity. Previously TGFβ was shown to induce collagen fibrillogenesis in rat an bovine AF cells in micromasses and electrospun scaffolds, respectively [15, 47]. Sheet formation only occurred in the presence of TGFβ, indicating the collagen maturation/fibrillogenesis largely depends on TGFβ signaling. Sheet formation in AF-S clones started approximately 4–5 days after TGFβ exposure and completely detached ECM sheets could always be observed at 6 days after TGFβ exposure. On average AF-nS clones initiated weak contraction (at which time ±10% of the surface was detached) of the ECM sheet at 5 days after start of TGFβ exposure and did not show detached cellular sheets before day 8 after start of TGFβ exposure. The clear correlation of sheet formation in AF-S clones with the expression of *P4HA1*, *P4HA2*, *P4HA3*, *PLOD1* and *LOX*, demonstrates their involvement in Collagen fiber formation and matrix contraction. Matrix contraction is rarely investigated: one study used smooth muscle cells in combination with a Collagen type I coating to induce matrix contraction within 24 hours [52]. MMP inhibition prevented matrix contraction while ascorbic acid and other anti-oxidants were unable to prevent contraction [52]. Hence, we speculate that sheet formation occurs rapidly, perhaps in conjunction with a certain critical Collagen fiber mass or cell density, under the influence of Collagen modifying enzymes. Interestingly ascorbic acid-induced sheet formation has only recently been reported in studies using mesenchymal stem cells [53]. These cell sheets were shown to promote the differentiation towards various lineages [54, 55]. An obvious caveat in the interpretation of these results is that mRNA levels do not necessarily reflect enzyme activity. Importantly, we here report clear differences in Collagen processing between AF subclones at the protein (*i*.*e*. functional) level.

In contrast to post-translational modification of proteoglycan molecules, which have been extensively studied in the IVD [13], relatively little is known with respect to post-translational modification of Collagen and its turnover in the intervertebral disc. The biochemical distribution of Collagen molecules varies massively depending on the location in the IVD (NP, inner AF, outer AF, CEP). It is conceivable that cells from the various layers of the AF differentially depend on their capacity to process Collagens for the maintenance of their specific surrounding matrix. A thorough study of Collagen post-translational modifications in the various compartments of the IVD might help provide possible answers.

## Conclusion

Structural integrity of the annulus fibrosus is a *condicio sine qua non* for intervertebral disc stability and function. Nevertheless research on low back pain and the process of disc degeneration has predominantly focused on the nucleus pulposus, as this is the tissue where the first signs of disc degeneration occur [56]. The importance of the annulus fibrosus for disc degeneration and pathologies was recently highlighted in several studies [6, 20]. In particular the AF cell phenotype remains largely undefined [21]. In this study we have generated the first immortal human AF cell lines that will enable studying AF cell biology with the advantages of a cell model. In addition, these clonogenic cells display differences in collagen fibril formation in the context of TGFβ exposure. The appearance of contracting collagen matrices in monolayer cultures correlates to expression of *P4HA1*, *P4HA3* and *LOX*. This difference in collagen fibril formation suggests differences in cell phenotype *in vitro*. Additional AF cell markers, preferably located at the cell surface to aid cell purification, are needed to further study and define the relevance of AF cell phenotypes *in vivo* and in disc degeneration. Increased understanding of AF cell biology will contribute to the development of novel approaches to treat AF defects and will further increase our understanding of IVD tissue homeostasis.

## Abbreviations

AC: articular cartilage
ACAN: Aggrecan
ADAMTS17: A disintegrin and metalloproteinase with thrombospondin motif, 17
AF: annulus fibrosus
AF-S: sheet-forming AF clone
AF-nS: non sheet-forming AF clone
bACT: beta Actin
BMP1: Bone morphogenetic protein 1
COL1/2/5/12A1: type I/II/V/XII Collagen alfa1
CEP: cartilaginous end plate
COMP: Cartilage oligomeric matrix protein
DDD: degenerative disc disease
CTRL: Control
ECM: extra cellular matrix
GAG: Glycosamine Glycan
hTERT: human Telomerase
iPSC: induced pluripotent stem cell
IVD: intervertebral disc
LOX: Lysyl oxidase
NC: notochordal (precursor) cells
NP: nucleus pulposus
P4HA1-3: Prolyl 4-hydroxylase, alpha polypeptide 1-3I
PDL: population doubling time
PLOD1-3: Procollagen-lysine, 2-oxoglutarate 5-dioxygenase 1–3
PPIB: Cylophillin B
ProCOL: Procollagen
RT-qPCR: real time quantitative polymerase chain reaction
SFRP2: Secreted frizzled-related protein
SOX9: SRY-box 9
SV40LTAg: Simian Virus 40 large T-antigen
TGFβ: Transforming growth factor beta
TLL1: Tolloid-like 1

## References

[1] Kroemer KE, Kroemer H, Kroemer-Elbert K. Skeletal Structures Engineering Physiology: Springer Berlin Heidelberg; 2010 p. 1–26.

[2] Adams MA, McNally DS, Dolan P. ‘STRESS’ DISTRIBUTIONS INSIDE INTERVERTEBRAL DISCS: THE EFFECTS OF AGE AND DEGENERATION. Journal of Bone & Joint Surgery, British Volume. 1996;78-B(6):965–72. 10.1302/0301-620x78b6.1287 8951017

[3] Eyre DR, Muir H. Types I and II collagens in intervertebral disc. Interchanging radial distributions in annulus fibrosus. Biochem J. 1976;157(1):267–70. Epub 1976/07/01. 962859 10.1042/bj1570267 PMC1163842

[4] Nosikova YS, Santerre JP, Grynpas M, Gibson G, Kandel RA. Characterization of the annulus fibrosus–vertebral body interface: identification of new structural features. Journal of Anatomy. 2012;221(6):577–89. 10.1111/j.1469-7580.2012.01537.x 22747710 PMC3512281

[5] Adams MA, Roughley PJ. What is intervertebral disc degeneration, and what causes it? Spine (Phila Pa 1976). 2006;31(18):2151–61. . 16915105 10.1097/01.brs.0000231761.73859.2c

[6] Lama P, Le Maitre CL, Dolan P, Tarlton JF, Harding IJ, Adams MA. Do intervertebral discs degenerate before they herniate, or after? Bone & Joint Journal. 2013;95-B(8):1127–33. 10.1302/0301-620x.95b8.31660 23908431

[7] Clouet J, Vinatier C, Merceron C, Pot-Vaucel M, Hamel O, Weiss P, et al The intervertebral disc: From pathophysiology to tissue engineering. Joint Bone Spine. 2009;76(6):614–8. 10.1016/j.jbspin.2009.07.002 19819178

[8] Christ B, Wilting J. From somites to vertebral column. Ann Anat. 1992;174(1):23–32. Epub 1992/02/01. . 1605355 10.1016/s0940-9602(11)80337-7

[9] Hayes AJ, Isaacs MD, Hughes C, Caterson B, Ralphs JR. Collagen fibrillogenesis in the development of the annulus fibrosus of the intervertebral disc. Eur Cell Mater. 2011;22:226–41. Epub 2011/11/04. vol022a18 [pii]. . 22048900 10.22203/ecm.v022a18

[10] Buckwalter JA. Aging and degeneration of the human intervertebral disc. Spine (Phila Pa 1976). 1995;20(11):1307–14. Epub 1995/06/01. . 7660243 10.1097/00007632-199506000-00022

[11] Choi K-S, Cohn MJ, Harfe BD. Identification of nucleus pulposus precursor cells and notochordal remnants in the mouse: Implications for disk degeneration and chordoma formation. Developmental Dynamics. 2008;237(12):3953–8. 10.1002/dvdy.21805 19035356 PMC2646501

[12] McCann MR, Tamplin OJ, Rossant J, SÃ©guin CA. Tracing notochord-derived cells using a Noto-cre mouse: implications for intervertebral disc development. Disease Models & Mechanisms. 2012;5(1):73–82. 10.1242/dmm.008128 22028328 PMC3255545

[13] Hayes AJ, Hughes CE, Ralphs JR, Caterson B. Chondroitin sulphate sulphation motif expression in the ontogeny of the intervertebral disc. Eur Cell Mater. 2011;21:1–14. Epub 2011/01/08. vol021a01 [pii]. . 21213210

[14] Bruehlmann SB, B. Rattner J, R. Matyas J, A. Duncan N. Regional variations in the cellular matrix of the annulus fibrosus of the intervertebral disc. Journal of Anatomy. 2002;201(2):159–71. 12220124 10.1046/j.1469-7580.2002.00080.x PMC1570900

[15] Hayes A, Ralphs J. The response of foetal annulus fibrosus cells to growth factors: modulation of matrix synthesis by TGF-Î^2^1 and IGF-1. Histochemistry and Cell Biology. 2011;136(2):163–75. 10.1007/s00418-011-0835-x 21739215

[16] Gruber HE, Hoelscher GL, Hanley EN Jr. Annulus cells from more degenerated human discs show modified gene expression in 3D culture compared with expression in cells from healthier discs. The Spine Journal. 2010;10(8):721–7. 10.1016/j.spinee.2010.05.014 20650410

[17] Gilbert H, Hoyland J, Freemont A, Millward-Sadler S. The involvement of interleukin-1 and interleukin-4 in the response of human annulus fibrosus cells to cyclic tensile strain: an altered mechanotransduction pathway with degeneration. Arthritis Research & Therapy. 2011;13(1):R8 10.1186/ar3229 21276216 PMC3241352

[18] Attia M, Santerre JP, Kandel RA. The response of annulus fibrosus cell to fibronectin-coated nanofibrous polyurethane-anionic dihydroxyoligomer scaffolds. Biomaterials. 2011;32(2):450–60. 10.1016/j.biomaterials.2010.09.010 20880584

[19] Jin L, Shimmer A, Li X. The challenge and advancement of annulus fibrosus tissue engineering. European Spine Journal. 2013:1–11. 10.1007/s00586-013-2663-2 PMC3657039 23361531

[20] Adams MA, Dolan P. Intervertebral disc degeneration: evidence for two distinct phenotypes. Journal of Anatomy. 2012;221(6):497–506. Epub 2012/08/14. 10.1111/j.1469-7580.2012.01551.x . 22881295 PMC3512277

[21] Guterl CC, See EY, Blanquer SB, Pandit A, Ferguson SJ, Benneker LM, et al Challenges and strategies in the repair of ruptured annulus fibrosus. European Cells & Materials. 2013;25:1–21. Epub 2013/01/04. 23283636 10.22203/ecm.v025a01 PMC3655691

[22] van den Akker G, Surtel D, Cremers A, Rodrigues-Pinto R, Richardson S, Hoyland J, et al Novel immortal human cell lines reveal subpopulations in the nucleus pulposus. Arthritis Research & Therapy. 2014;16(3):R135 10.1186/ar4597 PMC4227062 24972717

[23] Minogue BM, Richardson SM, Zeef LAH, Freemont AJ, Hoyland JA. Characterization of the human nucleus pulposus cell phenotype and evaluation of novel marker gene expression to define adult stem cell differentiation. Arthritis & Rheumatism. 2010;62(12):3695–705. 10.1002/art.27710 20722018

[24] Caron MMJ, Emans PJ, Coolsen MME, Voss L, Surtel DAM, Cremers A, et al Redifferentiation of dedifferentiated human articular chondrocytes: comparison of 2D and 3D cultures. Osteoarthritis and Cartilage. 2012;20(10):1170–8. 10.1016/j.joca.2012.06.016 22796508

[25] Prickaerts P, Niessen H, Mouchel-Vielh E, Dahlmans V, van den Akker G, Geijselaers C, et al MK3 controls Polycomb target gene expression via negative feedback on ERK. Epigenetics & Chromatin. 2012;5(1):12–24. 10.1186/1756-8935-5-12 PMC3499388 22870894

[26] Chou I, Reza A, Nicoll S. Distinct Intervertebral Disc Cell Populations Adopt Similar Phenotypes in Three-Dimensional Culture Tissue Engineering Part A. 2008;14(12):2079–87. 10.1089/ten.tea.2007.0337 18636941 PMC2809660

[27] Lee CR, Sakai D, Nakai T, Toyama K, Mochida J, Alini M, et al A phenotypic comparison of intervertebral disc and articular cartilage cells in the rat. Eur Spine J. 2007;16(12):2174–85. . 17786487 10.1007/s00586-007-0475-y PMC2140128

[28] Rutges J, Creemers LB, Dhert W, Milz S, Sakai D, Mochida J, et al Variations in gene and protein expression in human nucleus pulposus in comparison with annulus fibrosus and cartilage cells: potential associations with aging and degeneration. Osteoarthritis and Cartilage. 2009;18(3):416–23. 10.1016/j.joca.2009.09.009 19833252

[29] Minogue BM, Richardson SM, Zeef LA, Freemont AJ, Hoyland JA. Transcriptional profiling of bovine intervertebral disc cells: implications for identification of normal and degenerate human intervertebral disc cell phenotypes. Arthritis Res Ther. 2010;12(1):R22 10.1186/ar2929 20149220 PMC2875656

[30] Sakai DMDP, Nakai TB, Mochida JMDP, Alini MP, Grad SP. Differential Phenotype of Intervertebral Disc Cells: Microarray and Immunohistochemical Analysis of Canine Nucleus Pulposus and Anulus Fibrosus.: Spine 6 15, 2009;34(14):1448–1456; 2009. 10.1097/BRS.0b013e3181a55705 19525835

[31] Kluba T, Niemeyer T, Gaissmaier C, Grunder T. Human Anulus Fibrosis and Nucleus Pulposus Cells of the Intervertebral Disc: Effect of Degeneration and Culture System on Cell Phenotype. Spine. 2005;30(24):2743–8. 16371897 10.1097/01.brs.0000192204.89160.6d

[32] Wang J, Baer A, Kraus V, Setton L. Intervertebral Disc Cells Exhibit Differences in Gene Expression in Alginate and Monolayer Culture. Spine. 2001;26(16):1747–51. 11493844 10.1097/00007632-200108150-00003

[33] Lin Z, Willers C, Xu J, Zheng M-H. The Chondrocyte: Biology and Clinical Application. Tissue Engineering. 2006;12(7):1971–84. 10.1089/ten.2006.12.1971 16889526

[34] Clouet J, Grimandi G, Pot-Vaucel M, Masson M, Fellah HB, Guigand L, et al Identification of phenotypic discriminating markers for intervertebral disc cells and articular chondrocytes. Rheumatology. 2009;48(11):1447–50. 10.1093/rheumatology/kep262 19748963

[35] Sive JI, Baird P, Jeziorsk M, Watkins A, Hoyland JA, Freemont AJ. Expression of chondrocyte markers by cells of normal and degenerate intervertebral discs. Molecular Pathology. 2002;55(2):91–7. 10.1136/mp.55.2.91 11950957 PMC1187156

[36] Pattappa G, Li Z, Peroglio M, Wismer N, Alini M, Grad S. Diversity of intervertebral disc cells: phenotype and function. Journal of Anatomy. 2012;221(6):480–96. 10.1111/j.1469-7580.2012.01521.x 22686699 PMC3512276

[37] Lamplot JD, Liu B, Yin L, Zhang W, Wang Z, Luther G, et al Reversibly Immortalized Mouse Articular Chondrocytes Acquire Long-Term Proliferative Capability while Retaining Chondrogenic Phenotype. Cell transplantation. 2014 10.3727/096368914X681054 . 24800751

[38] Takezawa T, Mori Y, Yonaha T, Yoshizato K. Characterization of Morphology and Cellular Metabolism during the Spheroid Formation by Fibroblasts. Experimental Cell Research. 1993;208(2):430–41. 10.1006/excr.1993.1265 8375472

[39] Myllyharju J, Kivirikko KI. Collagens, modifying enzymes and their mutations in humans, flies and worms. Trends in Genetics. 2004;20(1):33–43. 10.1016/j.tig.2003.11.004 14698617

[40] Bailey AJ, Paul RG, Knott L. Mechanisms of maturation and ageing of collagen. Mechanisms of Ageing and Development. 1998;106(1–2):1–56. 10.1016/S0047-6374(98)00119-5 9883973

[41] Robertson WvB, Schwartz B. ASCORBIC ACID AND THE FORMATION OF COLLAGEN. Journal of Biological Chemistry. 1953;201(2):689–96. 13061407

[42] Berg RA, Prockop DJ. The thermal transition of a non-hydroxylated form of collagen. Evidence for a role for hydroxyproline in stabilizing the triple-helix of collagen. Biochemical and Biophysical Research Communications. 1973;52(1):115–20. 4712181 10.1016/0006-291x(73)90961-3

[43] Guillaume O, Daly A, Lennon K, Gansau J, Buckley SF, Buckley CT. Shape-memory porous alginate scaffolds for regeneration of the annulus fibrosus: effect of TGF-beta3 supplementation and oxygen culture conditions. Acta biomaterialia. 2014;10(5):1985–95. 10.1016/j.actbio.2013.12.037 . 24380722

[44] Roberts S, Evans EH, Kletsas D, Jaffray DC, Eisenstein SM. Senescence in human intervertebral discs. Eur Spine J. 2006;15 Suppl 3:S312–6. . 16773379 10.1007/s00586-006-0126-8 PMC2335374

[45] Gruber HE, Watts JA, Hoelscher GL, Bethea SF, Ingram JA, Zinchenko NS, et al Mitochondrial gene expression in the human annulus: in vivo data from annulus cells and selectively harvested senescent annulus cells. The Spine Journal. 2011;11(8):782–91. 10.1016/j.spinee.2011.06.012 21784712

[46] Estervig DN, Minoo P, Tzen CY, Scott RE. Three distinct effects of SV40 T-antigen gene transfection on cellular differentiation. J Cell Physiol. 1990;142(3):552–8. 10.1002/jcp.1041420314 . 2312615

[47] Lehmann HC, Chen W, Mi R, Wang S, Liu Y, Rao M, et al Human Schwann Cells Retain Essential Phenotype Characteristics After Immortalization. Stem Cells and Development. 2012;21(3):423–31. 10.1089/scd.2010.0513 . 21585251 PMC3272243

[48] Gruber H, Hoelscher G, Ingram J, Hanley E. Genome-wide analysis of pain-, nerve- and neurotrophin -related gene expression in the degenerating human annulus. Molecular Pain. 2012;8(1):63 10.1186/1744-8069-8-63 22963171 PMC3495673

[49] Occleston N, Laverty H, O'Kane S, Ferguson M. Prevention and reduction of scarring in the skin by Transforming Growth Factor beta 3 (TGFbeta3): from laboratory discovery to clinical pharmaceutical. J Biomater Sci Polym Ed. 2008;19(8):1047–63. 10.1163/156856208784909345 18644230

[50] de Clerck YA, Jones PA. The effect of ascorbic acid on the nature and production of collagen and elastin by rat smooth-muscle cells. Biochem J. 1980;186(1):217–25. Epub 1980/01/15. 7370010 10.1042/bj1860217 PMC1161522

[51] Schwarz RI, Mandell RB, Bissell MJ. Ascorbate induction of collagen synthesis as a means for elucidating a mechanism of quantitative control of tissue-specific function. Molecular and Cellular Biology. 1981;1(9):843–53. 10.1128/mcb.1.9.843 9279397 PMC369368

[52] Ivanov V, Roomi MW, Kalinovsky T, Niedzwiecki A, Rath M. Bioflavonoids effectively inhibit smooth muscle cell-mediated contraction of collagen matrix induced by angiotensin II. J Cardiovasc Pharmacol. 2005;46(5):570–6. Epub 2005/10/13. . 16220062 10.1097/01.fjc.0000179432.73007.45

[53] Wei F, Qu C, Song T, Ding G, Fan Z, Liu D, et al Vitamin C treatment promotes mesenchymal stem cell sheet formation and tissue regeneration by elevating telomerase activity. Journal of Cellular Physiology. 2012;227(9):3216–24. 10.1002/jcp.24012 22105792 PMC3299914

[54] Yu J, Tu Y-K, Tang Y-B, Cheng N-C. Stemness and transdifferentiation of adipose-derived stem cells using l-ascorbic acid 2-phosphate-induced cell sheet formation. Biomaterials. 2014;35(11):3516–26. 10.1016/j.biomaterials.2014.01.015 24462360

[55] Lui PPY, Wong OT, Lee YW. Application of Tendon-Derived Stem Cell Sheet for the Promotion of Graft Healing in Anterior Cruciate Ligament Reconstruction. The American Journal of Sports Medicine. 2014;42(3):681–9. 10.1177/0363546513517539 24451112

[56] Brayda-Bruno M, Tibiletti M, Ito K, Fairbank J, Galbusera F, Zerbi A, et al Advances in the diagnosis of degenerated lumbar discs and their possible clinical application. European Spine Journal. 2013:1–9. 10.1007/s00586-013-2960-9 23978994

